# CDH1 rs9929218 variant at 16q22.1 contributes to colorectal cancer susceptibility

**DOI:** 10.18632/oncotarget.9758

**Published:** 2016-06-01

**Authors:** Peng Han, Guiyou Liu, Xin Lu, Minmin Cao, Youling Yan, Jing Zou, Xiaobo Li, Guangyu Wang

**Affiliations:** ^1^ Department of Colorectal Surgery, The Affiliated Tumor Hospital of Harbin Medical University, Harbin, 150040, China; ^2^ Genome Analysis Laboratory, Tianjin Institute of Industrial Biotechnology, Chinese Academy of Sciences, Tianjin, 300308, China; ^3^ Department of Gastroenterology, The First Hospital of Harbin, Harbin, 150001, China; ^4^ Department of Endocrinology, The First Hospital of Harbin, Harbin, 150001, China; ^5^ Department of Hematology, The First Hospital of Harbin, Harbin, 150001, China; ^6^ Department of Pathology, Harbin Medical University, Harbin, 150081, China; ^7^ Department of Gastrointestinal Medical Oncology, The Affiliated Tumor Hospital of Harbin Medical University, Harbin, 150040, China

**Keywords:** genome-wide association study, colorectal cancer, rs9929218, meta-analysis, eQTL

## Abstract

Colorectal cancer (CRC) is the third most common cancer. Large-scale genome-wide association studies (GWAS) have been performed and reported some novel CRC susceptibility variants in European ancestry including the CDH1 rs9929218. Following GWAS and candidate studies evaluated the association between the CDH1 rs9929218 polymorphism and CRC in European, Asian and American populations. However, these studies reported inconsistent associations. Evidence shows that rs9929218 may regulate different gene expressions in different human tissues. Here, we reevaluated this association using large-scale samples from 16 studies (n=131768) using a meta-analysis method. In heterogeneity test, we did not identify significant heterogeneity among these studies. Meta-analysis using fixed effect model showed significant association between rs9929218 and CRC (*P*=6.16E-21, odds ratio (OR) =0.92, 95% confidence interval (CI) 0.91-0.94). In order to validate the effect of rs9929218 variant on CDH1 expression, we further performed a functional analysis using two large-scale expression datasets. We identified significant regulation relation between rs9929218 variant and the expression of CDH1, ZFP90, RP11-354M1.2 and MCOLN2 by both *cis-*effect and *trans-*effect. In summary, our analysis highlights significant association between rs9929218 polymorphism and CRC susceptibility.

## INTRODUCTION

Colorectal cancer (CRC) is the second leading cause of cancer death in developed countries [1]. It is a common complex human disease caused by the combination of genetic variants and environmental factors [2–3]. Large-scale genome-wide association studies (GWAS) and pathway analysis of GWAS datasets have been widely performed to identify common genetic variants or pathways contributing to human disease susceptibility [1, 4–15].

In 2008, Houlston et al. conducted a meta-analysis of two CRC GWAS including 13,315 individuals in discovery dataset and 27,418 subjects from eight independent case-control series in replication dataset [16]. They identified four new CRC risk variants at 14q22.2 (rs4444235, bone morphogenetic protein 4 (BMP4); *P* = 8.10E-10), 16q22.1 (rs9929218, cadherin 1 (CDH1); *P* = 1.20E-8), 19q13.1 (rrs9929218, rhophilin, Rho GTPase binding protein 2 (RHPN2); *P* = 4.60E-09) and 20p12.3 (rs961253; *P* = 2.00E-10) [16]. These findings underscore the value of large sample series for discovery and follow-up of genetic variants contributing to the etiology of CRC.

Followed the original CRC GWAS, several GWAS and candidate variant studies also investigate the association between rs9929218 and CRC in European, Asian and American populations [16–25]. However these studies reported consistent and inconsistent results. Evidence shows that rs9929218 variant may regulate different gene expressions in different human tissues, tumor tissues and normal tissues, and may have different regulatory mechanisms [26]. Here, we evaluated this association using large-scale samples from 16 studies (n=131768, 53656 CRC cases and 78112 controls) using meta-analysis method. To validate the effect of rs9929218 variant on CDH1 mRNA expression level, we further performed a functional analysis using two large-scale expression datasets.

## RESULTS

### Study selection

**Figure 1:**
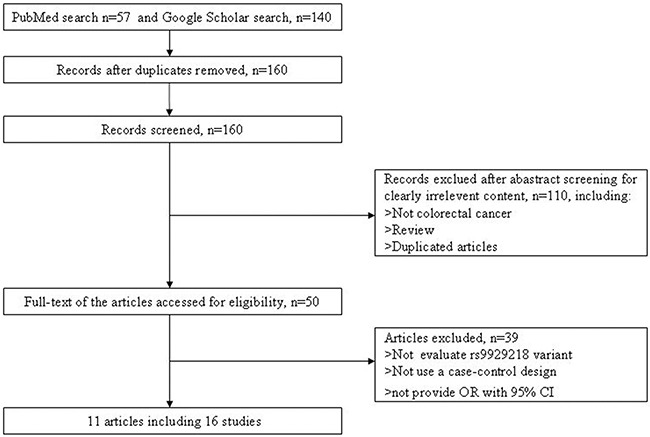
Flow diagram of selection of studies included in the current meta-analysis for the association between rs9929218 polymorphism and CRC

We selected 57 and 140 articles from PubMed and Google Scholar databases. Using the inclusion criteria and exclusion criteria, 11 articles including 16 independent studies were finally selected for our following analysis. More detailed information about the inclusion or exclusion of selected studies was described in Figure 1. The main characteristics of the included studies are described in Table 1.

**Table 1:** Main characteristics of selected studies in following meta-analysis

Study	Year	Ethnicity or country	Population	Case #	Control #	*P* value
Ho [17]	2011	China	Asian	892	890	0.965
Xiong [18]	2010	China	Asian	2124	2124	0.14
Li [50]	2012	China	Asian	229	267	0.484
Zhang [19]	2014	China, South Korea and Japan	Asian	14963	31945	0.03
He [20]	2011	European American	American	1171	1534	NA
He [20]	2011	African American	American	382	510	NA
He [20]	2011	Native Hawaiian	American	323	472	NA
He [20]	2011	Japanese Americans	American	1042	1426	NA
He [20]	2011	Latino	American	393	524	NA
Hutter [21]	2012	United States, Canada and Europe	American	7016	9723	0.01
Wang [22]	2013	African American	American	1894	4703	0.12
Kupfer [23]	2010	African American	American	795	985	0.44
Kupfer [23]	2010	European American	American	399	367	0.10
Mates [25]	2010	Romanian	European	92	96	0.303
Holst [24]	2010	Swedish	European	1755	1691	0.051
Houlston [16]	2008	United Kingdom	European	20186	20855	1.20E-08
			n =131768	n=53656	n=78112	

NA, not reported;

### Heterogeneity test

We evaluated the genetic heterogeneity using Cochran's Q statistic and *I*^2^ statistic, respectively. Using Cochran's Q statistic, we identified no significant heterogeneity among these 16 studies with Cochran's Q statistic = 17.62, and degrees of freedom = 15, and *P*=0.2829. We identified *I*^2^ = 14.9%, which indicates no statistically significant heterogeneity. We further performed a subgroup heterogeneity test in Asian, American and European populations, respectively. In the end, we found no significant genetic heterogeneity in Asian population with *I*^2^ = 0 and *P*=0.70, American population with *I*^2^ = 29% and *P*=0.19, and European population *I*^2^ = 11.2 and *P*=0.32.

### Meta-analysis

**Figure 2:**
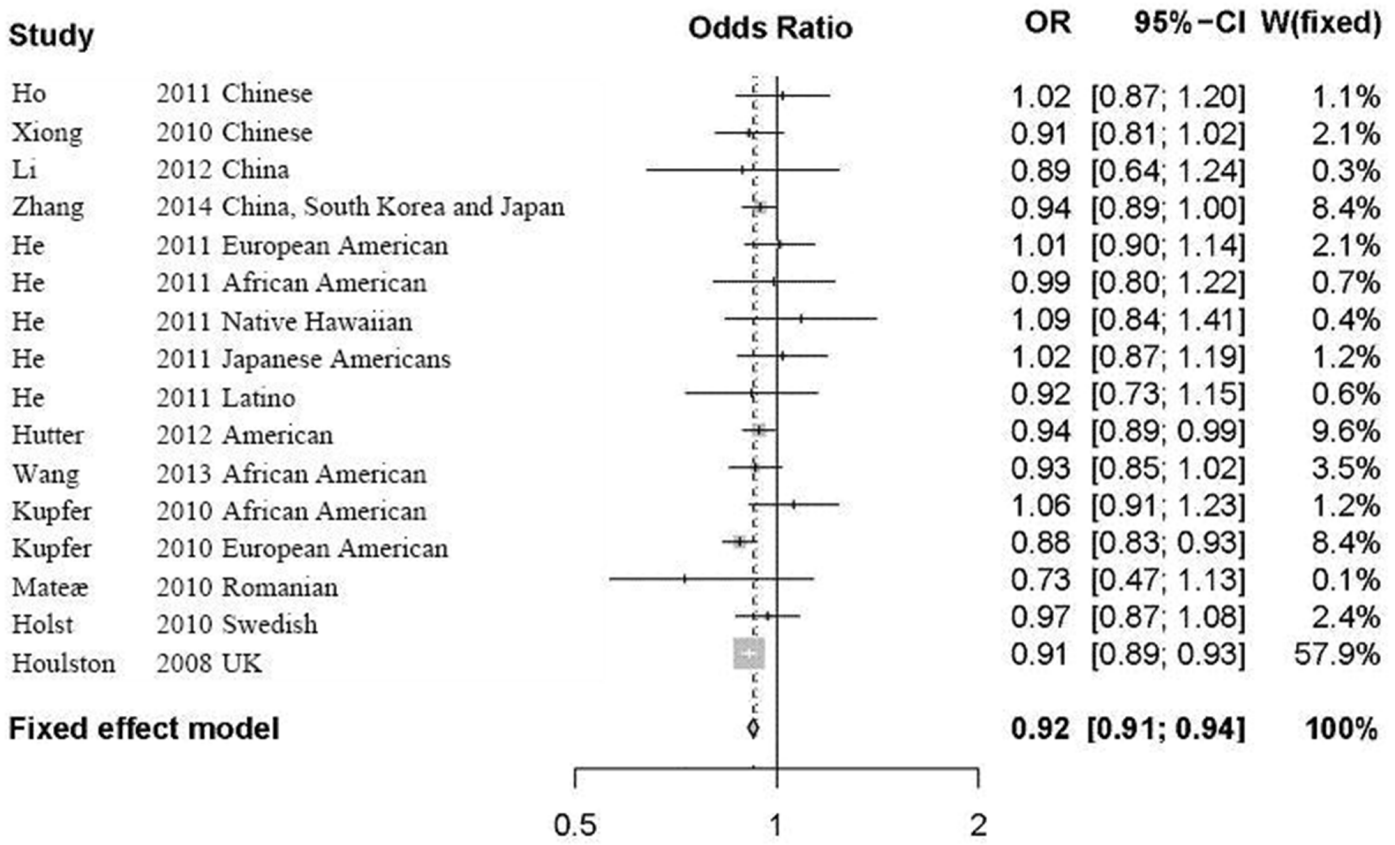
Forest plot for the meta-analysis of rs9929218 polymorphism using an additive model According to Table 1, 11 articles including 16 independent studies were finally selected for our following analysis. In Figure 2, we list the name of the first author, the year of publication, the population or ethnicity, the OR with 95% CI and the weight in meta-analysis. The overall OR was calculated by the fixed effect model. OR, odds ratio; CI, confidence interval; fixed, fixed effect model.

Considering no significant heterogeneity in all the selected studies, we calculated the overall OR by the fixed effect model. The meta-analysis results showed significant association between rs9929218 and CRC (*P*=6.16E-21, OR=0.92, 95% CI 0.91-0.94). Detailed results are described in Figure 2.

We further performed a subgroup meta-analysis in Asian, American and European populations, respectively. The results further supported the significant association between rs9929218 and CRC in Asian population (*P*=0.019, OR=0.94, 95% CI 0.90-0.99), American population (*P*=5.9E-05, OR=0.94, 95% CI 0.91-0.97), and European population (*P*=8.91E-17, OR=0.91, 95% CI 0.90-0.93).

**Figure 3:**
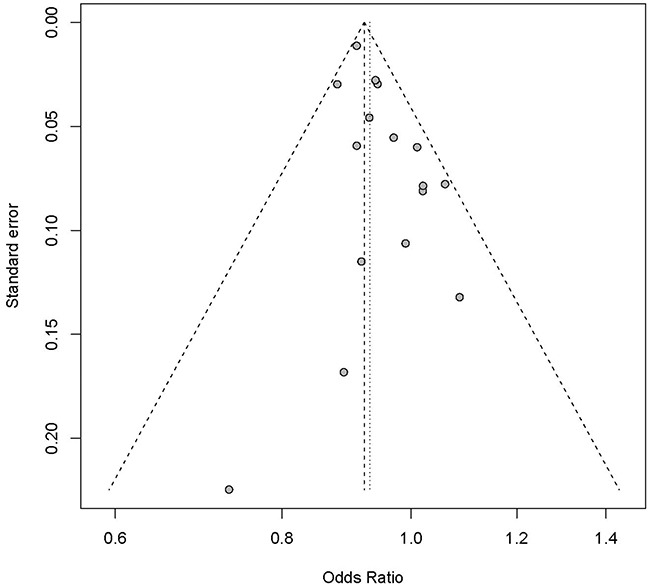
Funnel plot for publication bias analysis of the selected studies investigating the association between rs9929218 polymorphism and CRC The X-axis stands for the ORs and the Y-axis is the standard error for each of the 16 studies. A linear regression based approach proposed by Egger et al. is used to evaluate the asymmetry of the funnel plot.

The sensitivity analysis showed that the association between rs9929218 and CRC did not vary substantially. We also excluded the most two large-scale studies from Houlston [16] and Zhang [19] at a time. We observed no heterogeneity in other studies with *P*=0.3517 and *I*^2^ = 9.2%, and identified significant association between rs9929218 and CRC with *P*=1.79E-05. Evidence from the linear regression test suggests no significant publication bias with *P*= 0.03689. The funnel plot is a symmetrical inverted funnel as described in Figure 3.

### eQTL analysis

Using the eQTL dataset from the human peripheral blood, we identified that the rs9929218 minor allele (A) can significantly regulate ZFP90 expression with *P*=4.95E-152 by a *cis-*effect, and MCOLN2 expression with *P*=4.22E-06 by a *trans-*effect. Using GTEx, we further found that rs9929218 minor allele (A) can significantly regulate ZFP90 expression in the whole blood, Testis and Muscle-Skeletal tissues with *P*=6.60E-14, *P*=5.90E-06, and *P*=1.60E-05 by a *cis-*regulation. Meanwhile, rs9929218 can also regulate CDH1 expression in Nerve-Tibial and Spleen tissues with *P*=2.80E-11 and *P*=1.20E-06, respectively (Table 2)

**Table 2:** rs9929218 and gene expression in GTEx dataset

SNP	Gene Symbol	*P* value	Effect size	Tissue	Cis or trans
rs9929218	ZFP90	6.60E-14	−0.36	Whole Blood	*cis*
rs9929218	CDH1	2.80E-11	0.22	Nerve - Tibial	*cis*
rs9929218	CDH1	1.20E-06	−0.66	Spleen	*cis*
rs9929218	ZFP90	5.90E-06	0.46	Testis	*cis*
rs9929218	RP11-354M1.2	8.90E-06	−0.62	Spleen	*cis*
rs9929218	ZFP90	1.60E-05	−0.26	Muscle - Skeletal	*cis*

Effect size >0 and <0 mean that this SNP allele regulated increased and reduced gene expression, respectively.

## DISCUSSION

GWAS reported rs9929218 to be significantly associated with CRC in European ancestry [16]. Following studies investigated this association and reported inconsistent results. Here, using a comprehensive search in the PubMed and Google Scholar, we reevaluated this association using large-scale samples from 16 studies. Using two genetic heterogeneity test methods Cochran's Q-statistic and *I*^2^statistic, we did not identify significant heterogeneity among these 16 studies. Using a fixed effect model for meta-analysis to calculate the overall OR, we identified significant association between rs9929218 and CRC (*P*=6.16E-21, OR=0.92, 95% CI 0.91-0.94). The sensitivity analysis and publication bias further supported our results to be robust. After excluding the two large-scale studies from Houlston [16] and Zhang [19], we still identified significant association between rs9929218 and CRC.

Evidence shows that eQTL may modify gene expression and influence risk for human diseases [27–29]. In order to validate the effect of rs9929218 variant on CDH1 mRNA expression level, we performed eQTLs analysis using a large-scale dataset from the peripheral blood samples and multiple human tissues from GTEx. We identified significant regulation relations between rs9929218 variant and gene expression including CDH1, ZFP90, RP11-354M1.2 and MCOLN2 by both *cis-*effect and *trans-*effect. Recently, Closa et al. analyzed the association between genotypes for 26 GWAS SNPs and the expression of genes within a 2 Mb region (cis-eQTLs) using Affymetrix Human Genome U219 expression arrays with 47 samples from healthy colonic mucosa and 97 samples from normal mucosa adjacent to colon cancer [26]. In all the samples, the cis-eQTL analysis showed significant association between rs9929218 variant and GFOD2 expression with *P*=3.90E-03 [26]. Closa et al. further reported significant association of rs9929218 variant with expression of TSNAXIP1, HSF4 and NUTF2 in tumor tissue eQTL analysis with *P*=5.50E-03, *P*=6.70E-03 and *P*=1.70E-03, respectively, but not in normal tissue eQTL analysis with *P*=0.59, *P*=0.54 and *P*=0.27, respectively [26].

It is reported that the rs9929218 variant and rs1862748 are in linkage disequilibrium (LD) with rs28626308, which results in a non-synonymous change in RHPN2 protein [30]. In addition to rs9929218 variant, another two CDH1 variants rs4939827 and rs12953717 are in LD with rs7199991, which is genetically associated with four expression quantitative trait loci that correlate with expression of the upstream gene ZFP90 [30]. The rs9929218 is located at 16q22.1 (CDH1/ CDH3). Carvajal-Carmona et al. conducted a fine-mapping of CRC susceptibility loci at 8q23.3, 16q22.1 and 19q13.11 to refine the association signals [31]. Their findings suggest that ZFP90, rather than CDH1 or CDH3, is the most likely target of the 16q22.1 genetic variation associated with increased CRC risk [31].

Here, we reinvestigated the association between rs9929218 and CRC using an additive model as descried in Figure 2. It is reported that this model performs well when the true underlying genetic model is uncertain [32]. Analyzing the association between rs9929218 and CRC using dominant and recessive models is also required. However, the original genotype data are not publicly available. Future studies with genotype data are required to replicate our findings. In summary, our findings show that rs9929218 polymorphism contributes to CRC susceptibility.

## METHODS AND MATERIALS

### Search strategy

We searched the PubMed database to select all possible studies with the combined key words including ‘CDH1′, ‘rs9929218′, ‘polymorphism’, or ‘variant’ and ‘colorectal cancer’, ‘colon cancer’, ‘rectal cancer’, or ‘bowel cancer’. We also used the Google Scholar to query the articles citing CRC GWAS and select all associated publications. There was no language and sample limitation, and the literature search was updated on October 28, 2015. This search was performed by two independent individuals to identify potential publications meeting our inclusion criteria (see below), with differences resolved by discussion with a third reviewer. More detailed information about the search strategy is described in previous studies [33–35].

### Inclusion and exclusion criteria

The following inclusion criteria were used to select all potential publications for the meta-analysis: (1) use a case-control design; (2) evaluate the association between rs9929218 and CRC; (3) provide odds ratio (OR) with 95% confidence interval (CI) for allele model (see below); or (4) provide sufficient data to calculate the OR and 95% CI for allele model; For any publication, it is excluded from the meta-analysis, if (1) not for cancer research; (2) not use a case-control design including case population; case reports, conference abstract, reviews; (3) not provide OR with 95% CI for allele model (see below); or (4) not provide sufficient data to calculate the OR and 95% CI for allele model; More detailed information is described in previous studies [33–35].

### Data extraction

Two authors reviewed and extracted information from all eligible publications independently, according to the inclusion and exclusion criteria listed above. The following items were extracted including (1) the name of the first author; (2) the year of publication; (3) the population and ethnicity; (4) the numbers of CRC cases and controls; (5) the OR with 95% CI or (6) to calculate the OR and 95% CI; If there is a conflict, both reviewers discuss with third reviewer and reach an agreement. For rs9929218 polymorphism A/G alleles, we selected the additive genetic model: A allele versus G allele for further meta-analysis [36–40].

### Heterogeneity test

Genetic heterogeneity is evaluated using two methods including Cochran's Q-statistic and I2=(Q−(k−1))Q×100% statistic [41–46]. Cochran's Q statistic approximately follows a *χ*^2^ distribution with k-1 degrees of freedom (k stands for the number of studies for analysis) [41–46]. A significant Q-statistic (*P* < 0.01) indicates heterogeneity among selected studies. *I*^2^ is a measure of heterogeneity and a statistic that indicates the percentage of variance in a meta-analysis that is attributable to study heterogeneity [41–46]. The intervals including 0-25%, 25-50%, 50-75% and 75-100%, represent the low, moderate, large and extreme heterogeneity [41–46]. The interval *I*^2^ > 50% indicates statistically significant heterogeneity.

### Statistical analysis

Here, we used two kinds of meta-analysis models including the fixed effect model (Mantel-Haenszel) and random-effect model (DerSimonian-Laird) to calculate the pooled OR. If there is no significant heterogeneity among the included studies, the pooled OR is calculated by the fixed effect model; otherwise the OR is calculated by random-effect model [41–46]. Z test is used to determine the significance of OR. R Package is used to compute all statistical tests [41–46]. A significant levels (*P* < 0.01) indicates significant association between rs9929218 and CRC. Using sensitivity analysis, each study is omitted at a time to assess the influence of single study on the association between rrs9929218 and CRC. A funnel plot proposed by Egger et al. is used to investigate potential publication bias [41–46]. A linear regression based approach proposed by Egger et al. is used to test for publication bias, and provide statistical evidence to evaluate the asymmetry of the funnel plot to. with the *P* < 0.01 indicating that there was a significant publication bias [47]. All statistical analyses were performed using R Package [41–46].

### eQTL analysis in human peripheral blood

To validate the effect of rs9929218 variant on CDH1 mRNA expression level, we performed an expression quantitative trait loci (eQTL) analysis using a large-scale eQTL dataset, which is from a meta-analysis in non-transformed peripheral blood samples from 5,311 individuals with replication in 2,775 individuals [48].

### eQTLs analysis using Genotype-Tissue Expression

We also investigate the effect of rs9929218 variant on CDH1 mRNA expression using the Genotype-Tissue Expression (GTEx) project (http://www.gtexportal.org/home/), which provides a scientific resource to study human gene expression and regulation and its relationship to genetic variation [49]. Here, we selected all the human tissues with the number of genotyped samples > 60 in the following eQTL analysis [49].
